# Miscellaneous Surgical Essays

**Published:** 1781-08

**Authors:** 


[ *To be continued.* ]

---

V. *Miſcellaneous ſurgical eſſays, by* J. L. Schmucker, Vol. II. *(Continued from page 20.)*

XIV. A *Caſe in which a portion of the thigh bone was extracted from an abſceſs.. By* Mr. Horn.—The abſceſs here ſpoken of came on after a quartan. It ſuppurated and was opened, but for a conſiderable length of time ſhewed no diſpoſition to heal. At length a portion of bone exfoliated and was extracted, ſoon after which a cure was effected. The patient informed our author, that thirteen years before the appearance of this abſceſs he had hurt his thigh by a fall, which had left a ſwelling that was occaſionally attended with pain, ſo that the whole of his complaint probably aroſe from that accident.

XV. *Obſervations on the abuſe of blood-letting in forced marches. By* the ſame.—The author remarks, that venæſection often proves fatal when practiſed after a forced march, and at a time when the ſoldier, fatigued by the heat of the ſeaſon and climate, and, worn down with fatigue, fatigue, is ſinking under the weight of his baggage. He tells us, he has often met with inſtances of this ſort. The countenance of the patient was generally pale, his eyes prominent, his voice, reſpiration, and pulſe weak and ſometimes hardly perceptible. Some were delirious, and theſe he obſerves commonly died. If they were blooded the blood came away by drops, and the evacuation generally proved fatal.

The mode of treatment recommended by Mr. Horn in theſe caſes is as follows: he directs the patients to be laid in a cool ſituation, with their head and breaſt a little elevated. He then adviſes vinegar (or, if that cannot be had, ſpirit of vitriol diluted with water) to be given them to drink. At the ſame time the hands and face of ſuch as are ſenſeleſs are to be bathed with vinegar, and volatile alkali applied to their noſtrils. If this method ſhould fail to rouſe them the internal membrane of the noſe is to be lightly touched with a ſtraw, till a ſneezing is excited, after which we are told that in general the patient gradually recovers, and it is then adviſeable for him to march on again as ſoon as poſſible.

Mr. Horn obſerves, that many were prevented from falling, by giving them acidulated drink and and allowing them a little reſt as ſoon as they began to be affected.

XVI. *Caſe of a caries of the os frontis. By the ſame.*—This diſeaſe was owing to a venereal taint. There were two fiſtulous openings in the forehead, which afforded an ichorous diſcharge, but the integuments were neither diſcoloured nor painful, ſo that the patient wore his hat as uſual without any inconvenience. He died of a fever, and on diſſection our author found the outer table of the os frontis intirely conſumed and the ſinus frontales obliterated. This caſe is a curious confirmation of what has been more than once obſerved with reſpect to the almoſt imperceptible progreſs of a venereal caries.

XVII. *Caſe of an inverted ſcirrhous uterus. By the ſame.* This complaint was the conſequence of delivery. The inverted uterus remained for a long time in the vagina, but at length prolapſed, and was then found to be ſcirrhous. Its bulk was equal to that of a child's head, and from its ſurface, which had ſeveral ſcirrhous excreſcences, an acrid fœtid mucus was perpetually exſuding. At length it became gangrenous and the patient died.

XVIII. *Caſe of a gun-ſhot wound. By* Mr. Völker.—In this caſe the ball penetrated into the the abdomen, and paſſed, as appeared afterwards, near the left kidney. Violent ſymptoms enſued; the patient complained of a burning pain in the neighbourhood of the ſpine, and the wound diſcharged only a fœtid ichor. On the twenty-fourth day after the accident, the ball was diſcharged by ſtool, and all the ſymptoms were mitigated. On the thirty-ſixth day, however, they returned with increaſed violence, but were again relieved by the diſcharge of ſeveral pieces of cloth from the wound. After this the patient's life was a third time endangered by an error in diet, which occaſioned violent ſpaſms of the inteſtines. A diarrhœa relieved him from theſe alarming ſymptoms, and he afterwards gradually recovered.

XIX. *An account of the amputation of an arm after a gun-ſhot wound, by* Mr. Budæus.—In the caſe here related the patient's ſtrength was ſo much reduced by the profuſe ſuppuration occaſioned by the wound, that it was feared he would not ſurvive the amputation of his arm. Happily, however, ſoon after the operation the hectic fever ſubſided, and by means of a nouriſhing diet the patient recovered in a ſhort time.

XX. *Remarks on the efficacy of topical bleeding in local inflammations of the head, by* Mr. Sellie.—

As a proof of the good effects of this practice the author ſpeaks of a wound of the head, attended with ſtupor, in which the trepan was applied, and the veſſels of the dura mater being found extremely diſtended with blood, an inciſion was made into them, and the patient ſoon afterwards recovered his ſenſes. Mr. Sellie mentions another caſe, which deſerves to be noticed, on account of a ſingular circumſtance that attended it. It ſeems that on applying the trepan to a patient's ſcull, the inſtrument made its way through the bone much ſooner than was expected, and wounded the brain. On inquiry it was found that this part of the cranium was carious. Notwithſtanding this dangerous accident, however, the patient got well.

XXI. *On the efficacy of Peruvian bark in a caſe of abſceſs attended with a caries of the os humeri, by* the ſame.——The abſceſs here deſcribed came on after a putrid fever, and produced a caries of the head of the os humeri and of the proceſſes of the ſcapula, by which the capſular ligament and ſeveral muſcles were deſtroyed, ſo that the cavity of the joint became expoſed. The patient laboured at the ſame time under hectic fever, colliquative ſweats, and diarrhœa. In this alarming ſtate our author adminiſtered the bark, and and in the ſpace of ſix weeks had the pleaſure of ſeeing his patient perfectly recovered.

XXII. *Caſe of a mortification of the fore-arm, by* Mr. Morgenſtern. In this caſe the patient's arm was at firſt cold and without pulſe. Theſe ſymptoms were followed by violent pain and inflammation, which terminated in a mortification. The limb was amputated about the middle of the os humeri, and upon diſſection the three principal branches of the humeral artery were found completely obſtructed by a dry polypous concretion of a dark red colour, ſo that not a drop of blood could paſs through them. It appeared that ſome time before this the patient had had a fractured clavicle, the callus of which on examination ſeemed to be very irregular, and that end of it which is attached to the acromion was depreſſed.

XXIII. *Caſe of a cancerous clitoris, by* Mr. Kramer.—The clitoris in this caſe was equal to three fingers in thickneſs, and an inch in length. At its fore part it was in a ſtate of ulceration, and covered with cancerous excreſcences, ſo as to reſemble the head of a cauliflower. The left nympha was free from diſeaſe, but the right was much indurated. An attempt was firſt made to remove the clitoris by ligature; but this me thod proved ſo extremely painful that our author found it neceſſary to have recourſe to the knife, with which he extirpated it completely, together with the ſchirrous portion of the diſeaſed nympha. The hæmorrhage was eaſily ſuppreſſed by the application of agaric, and the cure accompliſhed without any bad ſymptoms.

XXIV. *Caſe of an aneuriſm, by* the ſame.— In this caſe the coats of the diſeaſed veſſel burſt, but the hæmorrhage was ſtopt by means of the tourniquet, after which the artery was ſecured by a ligature, and a few hours after the operation the pulſe was felt in the wriſt, ſo that the patient ſeemed to be in a fair way of recovery. Unexpectedly, however, a locked jaw took place on the fifth day, and in two hours deſtroyed the patient. The author aſcribes this fatal ſpaſm to the irritable habit of the patient, and aſſures us, that he had not included the nerve in the ligature.

[ *To be continued.* ]

SECTION

